# Cell-to-cell transfer of SAA1 protein in a cell culture model of systemic AA amyloidosis

**DOI:** 10.1038/srep45683

**Published:** 2017-03-31

**Authors:** Stephanie Claus, Ioana Puscalau-Girtu, Paul Walther, Tatiana Syrovets, Thomas Simmet, Christian Haupt, Marcus Fändrich

**Affiliations:** 1 Institute of Protein Biochemistry, Ulm University, Helmholtzstr. 8/1, 89081 Ulm, Germany; 2 Central Facility for Electron Microscopy, Ulm University, Albert-Einstein-Allee 11, 89069 Ulm, Germany; 3 Institute of Pharmacology of Natural Products and Clinical Pharmacology, Ulm University, Helmholtzstr. 20, 89081 Ulm, Germany

## Abstract

Systemic AA amyloidosis arises from the misfolding of serum amyloid A1 (SAA1) protein and the deposition of AA amyloid fibrils at multiple sites within the body. Previous research already established that mononuclear phagocytes are crucial for the formation of the deposits *in vivo* and exposure of cultures of such cells to SAA1 protein induces the formation of amyloid deposits within the culture dish. In this study we show that both non-fibrillar and fibrillar SAA1 protein can be readily transferred between cultured J774A.1 cells, a widely used model of mononuclear phagocytes. We find that the exchange is generally faster with non-fibrillar SAA1 protein than with fibrils. Exchange is blocked if cells are separated by a membrane, while increasing the volume of cell culture medium had only small effects on the observed exchange efficiency. Taken together with scanning electron microscopy showing the presence of the respective types of physical interactions between the cultured cells, we conclude that the transfer of SAA1 protein depends on direct cell-to-cell contacts or tunneling nanotubes.

AA amyloidosis is a classical form of systemic amyloidosis that involves amyloid deposits in multiple organs^1^. The disease usually affects spleen, liver and kidneys and shows a worldwide distribution in humans^2^. It occurs in several other mammalian species as well as in birds^3^, being thus similar to prion diseases, for which more than 50 mammalian species are susceptible^4^. Underlying causes of AA amyloidosis are chronic inflammatory disorders or infections, such as rheumatoid arthritis or familial Mediterranean fever^5^. AA amyloid fibrils consist of AA protein, which represents in humans and in mice an N-terminal fragment of the SAA1 protein^6^. Globular SAA1 adopts a four-helix bundle conformation and belongs to the all-alpha class of proteins^7^. Lipid-free SAA1 that is kept at 4 °C *in vitro* is prone to self-assemble into α-helical hexamers or other oligomers^8^, which contrast to the β-sheeted structure adopted in the amyloid fibril.

SAA1 is an extracellular acute-phase protein that circulates within the blood normally at a concentration of 1–2 μg/ml^9^. In response to a strong inflammatory stimulus, however, its serum concentrations become dramatically upregulated to ultimately reach levels of more than 1 mg/ml^6^. While the native function of SAA1 is not finally established, the protein can interact with macrophages and modulates their lipid homeostasis in the course of an inflammation^10^. Macrophages can internalize SAA1 protein or AA amyloid fibrils^11^,^12^,^13^ and are involved in the degradation and clearance of amyloid deposits^13^ as well as in the biogenesis of amyloid deposits *in vivo*^14^. Macrophages are associated with tissue-amyloid^15^ and depletion of these cells with clodronate-containing liposomes antagonizes the development of AA amyloidosis in mice^14^,^16^.

Exposure of cultures of primary monocytes, macrophages or monocytic cell lines to acute-phase levels of SAA1 protein in the medium leads to the formation of amyloid deposits within the culture dish and has given rise to a facile cell culture model for studying the process of cellular amyloid biogenesis^17^,^18^. The resulting amyloid deposits show classical features of tissue-deposited amyloid^12^,^19^, such as the presence of glycosaminoglycans, lipids and serum amyloid P component^20^,^21^,^22^. The cell model has previously been used to study the effect of natural modulators of fibril formation, such as proteases^23^, or the network assembly of the fibrils constructing an amyloid deposit^22^. In this study it is used to address the question of a possible cell-to-cell transfer of soluble SAA1 protein and SAA1 fibrils.

This issue has so far been analysed for several neurodegenerative amyloid diseases where the transfer of amyloid proteins or aggregates is thought to underlie the spreading of disease inside the brain^24^. While systemic AA amyloidosis is arguably the best candidate of a mammalian prion disease that does not involve the prion protein^25^ much less is known about the relevance of cell-to-cell transfer in systemic amyloidosis. Long-standing evidence shows injection of spleen homogenates or extracts from AA amyloidotic mice into inflamed recipient animals leads to the rapid development of AA amyloidosis^26^,^27^. The molecular agent underlying this activity has been termed ‘amyloid enhancing factor’ (AEF)^28^ and was suggested to represent a prion-like agent and to consist mainly of AA amyloid fibrils^29^. AEF promotes the deposition of amyloid from SAA1 protein in the cell culture model^18^,^20^, and there is evidence for the spreading of AA amyloid in humans and in mice^3^,^30^,^31^, although the cellular basis of these reactions remained elusive. In this study we now address this issue and analysed the possible transfer of SAA1 fibrils and non-fibrillar SAA1 protein in the cell culture model of systemic AA amyloidosis.

## Results

### Intracellular SAA1 protein is transferred from cell to cell

**Figure 1:**
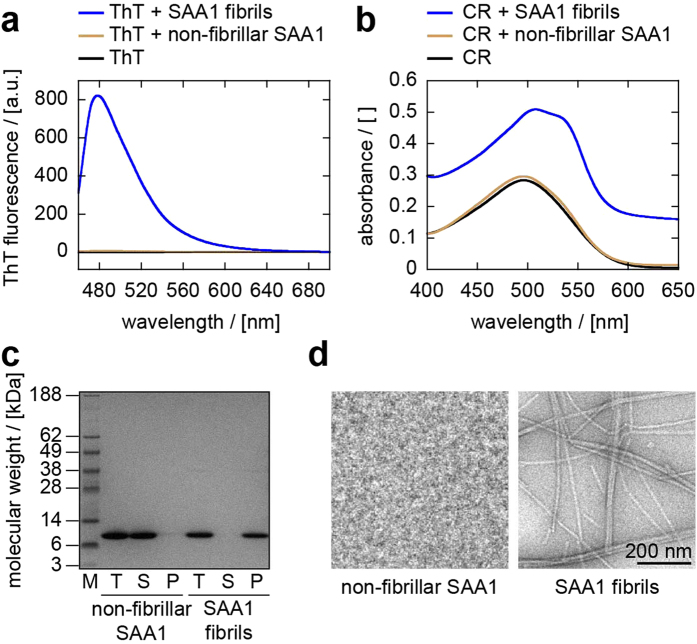
Analysis of SAA1 fibrils and non-fibrillar SAA1. (**a**,**b**) ThT fluorescence (**a**) and CR absorption (**b**) spectra of SAA1 fibrils (blue), freshly dissolved, non-fibrillar SAA1 protein (ochre) and of dye in buffer (black). (**c**) Coomassie-stained LDS-PAGE gel of non-fibrillar SAA1 and SAA1 fibrils before and after centrifugation for 30 min at 16,000 *g* at 4 °C. M: molecular weight marker; T: total sample before centrifugation; S: supernatant; P: pellet after centrifugation resuspended in the original volume. (**d**) Negative stained TEM images of freshly dissolved SAA1 protein and SAA1 fibrils.

To test for a possible cell-to-cell transfer of SAA1 protein, we analysed the fate of two different forms of SAA1 in the cell model, SAA1 fibrils and non-fibrillar SAA1 protein. SAA1 fibrils refer to samples of aged recombinant SAA1 protein that bind the amyloid-binding dyes Thioflavin T (ThT; Fig. 1a) and Congo red (CR; Fig. 1b) and contain SAA1 protein that can quantitatively be pelleted by centrifugation for 30 min at 16,000 *g* (Fig. 1c). Consistent with the presence of amyloid-like structures, we found large quantities of fibrils with transmission electron microscopy (TEM; Fig. 1d). The second protein form, non-fibrillar SAA1, essentially represents samples of freshly dissolved recombinant SAA1 protein that do not bind ThT or CR and where the protein remains soluble after centrifugation (Fig. 1c). We further confirmed the absence of fibrillar structures in these samples by using TEM, indicating the presence of monomeric and low-oligomeric aggregation species (Fig. 1d).

**Figure 2:**
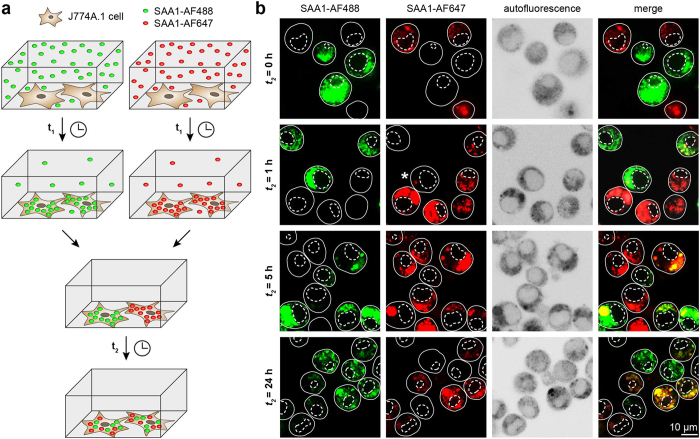
LSM shows the propagation of non-fibrillar SAA1 from cell to cell. (**a**) Schematic representation of the experimental outline. J774A.1 cells were incubated with 1 mg/ml non-fibrillar unlabelled SAA1 and 0.02 mg/ml fluorescently labelled SAA1 (either SAA1-AF488 or SAA1-AF647 protein) for an initial time period of *t*_*1*_ = 24 h. The supernatant was taken off and the cells were mixed in 1:1 ratio for a further incubation step of variable duration (*t*_*2*_) in which no SAA1 was added to the supernatant. (**b**) LSM images of cells co-cultured for different time periods *t*_*2*_ according to panel a. Different filter settings were used to visualize the distribution of SAA1-AF488 and SAA1-AF647 proteins. The autofluorescence image shows the cell boarder (continuous line) and nucleus (dotted line). White asterisk in the second row from the top highlights a double positive cell.

We then tested as to whether or not non-fibrillar SAA1 can be transferred from cell to cell. To that end we used SAA1 protein that was N-fluorescently labelled with Alexa Fluor 488 (AF488) or Alexa Fluor 647 (AF647) dyes. These protein forms, termed SAA1-AF488 or SAA1-AF647 were added at 0.02 mg/ml concentration, together with 1 mg/ml non-labelled SAA1 (all proteins were freshly dissolved) to murine macrophage-like J774A.1 cells such that the cells were loaded with the fluorescent protein for an initial time period *t*_*1*_ of 24 h. Any remaining extracellular SAA1 protein was removed by mild trypsination and the cells were collected by scraping. Cells loaded with SAA1-AF488 were mixed with those loaded with SAA1-AF647 and transferred to new plates to enable their further co-incubation for a time period *t*_*2*_ (Fig. 2a). Monitoring this mixture of cells with laser scanning microscopy (LSM) revealed cells at time point *t*_*2*_ = 0 h to be positive for either SAA1-AF488 or SAA1-AF647 protein but not for both labelled protein variants simultaneously (Fig. 2b, first row). Extending the *t*_*2*_ incubation period to 1 h revealed that only few cells were positive for both fluorescent protein variants (Fig. 2b, second row), while extending the co-incubation period *t*_*2*_ to 24 h dramatically increased the proportion of these cells. Both fluorescent protein variants could now be detected in most cultured cells, suggesting their transfer from cell to cell (Fig. 2b, last row).

**Figure 3:**
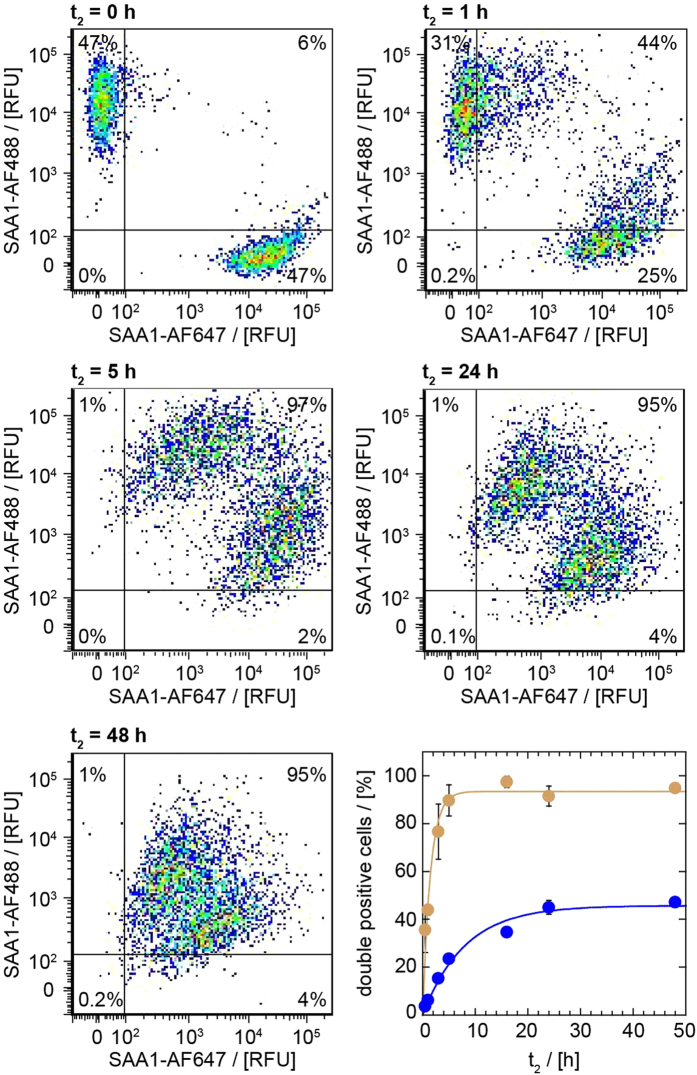
Flow cytometry shows the cell-to-cell propagation of non-fibrillar and fibrillar SAA1. Flow cytometric analysis of J774A.1 cells, which were preincubated for a time period *t*_*1*_ of 24 h with 1 mg/ml non-labelled SAA1 and 0.02 mg/ml fluorescently labelled SAA1 (either SAA1-AF488 or SAA1-AF647), and co-cultured for different periods of time (*t*_*2*_) as indicated in the figure. The vertical and horizontal lines represent the thresholds above cells that were considered as SAA1-AF488 and SAA1-AF647 positive. The lower right panel shows a quantification of the cell-to-cell transfer as taken from percent of cells within the upper right quadrant (double positive cells) of the scatter plot of SAA1-AF488 vs. SAA1-AF647 fluorescence (n = 3). Ochre data points represent cells incubated with non-fibrillar SAA1 and blue ones represent cells incubated with fibrillar SAA1. The data were fitted using a mono-exponential function. Error bars may be smaller than the symbol size. RFU: relative fluorescence unit.

Fully consistent results were obtained when monitoring the exchange with flow cytometry. Only 6% of the cells contained both labelled SAA1 proteins (SAA1-AF488 and SAA1-AF647) by this method at time point *t*_*2*_ = 0 h (Fig. 3, first row, left), suggesting, however, some exchange to have taken place in the dead time of the experiment or during sample work up. By contrast, the percentage of double positive cells significantly became increased to 44% as the t_2_ incubation period was extended to 1 h (Fig. 3, first row, right) and a value of 91% was reached upon prolonging t_2_ to 24 h (Fig. 3, middle row, right). We could fit the temporal development of double positive cells to the mono-exponential function


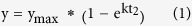


in which *y* represents the percentage of double fluorescent cells, *y*_*max*_ the maximum value of *y* reached in the time course of this experiment, *k* the rate constant of the formation of double positive cells and *t*_*2*_ the co-incubation time period. The values of *k* and *y*_*max*_ obtained with non-fibrillar SAA1 were 0.68 h^−1^ and 93% respectively. At variance to non-fibrillar SAA1, the same experiment performed with a combination of two cell populations that had separately been loaded with differently labelled SAA1 fibrils, yielded a rate constant of only 0.13 h^−1^ and a *y*_*max*_ value of 46%. That is, the exchange of non-fibrillar SAA1 was much faster than that of SAA1 fibrils and also reached a higher *y*_*max*_ value within the analysed period of time. In other words, the transfer efficiency decreases as the protein size is increased. Nevertheless, both tested forms of SAA1 protein become efficiently transferred between J774A.1 cells.

### Exchange of SAA1 is blocked if cell-to-cell contacts are prevented

**Figure 4:**
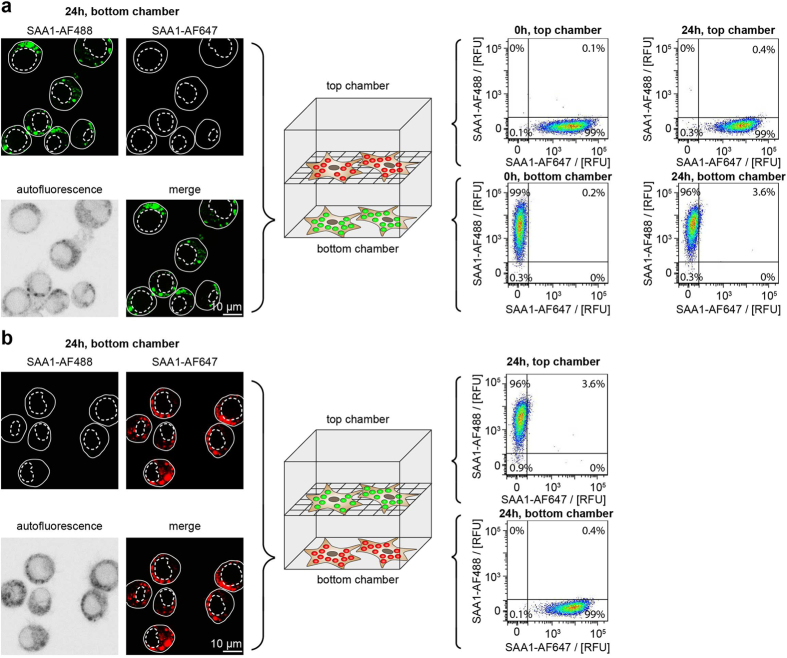
Preventing direct cell-to-cell contacts decreases the transfer efficiency of non-fibrillar SAA1. (**a**) LSM (left) and flow cytometric (right) analysis of J774A.1 cells that were preincubated for *t*_*1*_ = 24 h with 1 mg/ml non-fibrillar non-labelled SAA1 and 0.02 mg/ml fluorescently labelled SAA1 (either SAA1-AF488 or SAA1-AF647) and further incubated for 0 h or 24 h (*t*_*2*_) in one culture plate but separated by a 0.4 μm membrane. The top chamber contains cells that had internalized SAA1-AF647, the bottom chamber contains cells that had internalized SAA1-AF488. Different filter settings were used to visualize the distribution of SAA1-AF488 and SAA1-AF647 by LSM. The autofluorescence LSM image was used to localize the cell boarder (continuous line) and nucleus (dotted line). (**b**) LSM (left) and flow cytometric (right) analysis of cells incubated as described in (**a**) except that the cells in the top chamber had internalized SAA1-AF488, while the cells in the bottom chamber had internalized SAA1-AF647 protein. RFU: relative fluorescence unit.

We then tested as to whether or not direct physical contacts between the cells might be necessary for this exchange to occur or whether transfer takes place mainly through the medium. We therefore co-incubated J774A.1 cells that had separately been loaded with either 0.02 mg/ml SAA1-AF488 and 1 mg/ml non-labelled SAA1 or 0.02 mg/ml SAA1-AF647 and 1 mg/ml non-labelled SAA1 (all of the proteins were added in their non-fibrillar form) for a time period *t*_*2*_ of 24 h. In contrast to the experimental conditions used in Fig. 2a, we this time separated the two cell populations by a 0.4 μm membrane which prevented the formation of direct interactions between the cultured cells, whilst retaining the possibility of an exchange between the two chambers through the medium (Fig. 4). LSM analysis of cells kept under these conditions revealed that the vast majority of the cells in the bottom chamber contained only that form of fluorescent SAA1 protein with which the cells were loaded; that is, cells loaded with SAA1-AF488 contained almost exclusively SAA1-AF488 after incubation (Fig. 4a, left), while cells loaded with SAA1-AF647 contained mostly SAA1-AF647 protein after incubation (Fig. 4b, left). However, almost none of the bottom cells had adopted the SAA1 variant from the top chamber.

Consistent data were obtained by flow cytometry, which enabled us to analyse the cells from top and bottom chamber. 96% of the bottom chamber cells that had been loaded with SAA1-AF488 did not comprise any SAA1-AF647 after *t*_*2*_ = 24 h (Fig. 4a, right). 99% of the top chamber cells that were initially loaded with SAA1-AF647 protein lacked the other protein variant at the end of the co-incubation period (Fig. 4a, right). Analogous results were obtained if SAA1-AF488 and SAA1-AF647 loaded cells were flipped between bottom and top chamber (Fig. 4b, right). In a control experiment, we ensured that SAA1 was able to pass through the membrane. In this experiment we added 1 mg/ml SAA1 protein to the medium of the bottom chamber in the absence of any cells. After an incubation of 24 h the protein composition was analysed in the top and bottom chamber medium by denaturing protein gel electrophoresis. We found equal concentrations of SAA1 in both compartments, indicating that the protein had passed through the membrane (Fig. S1).

**Figure 5:**
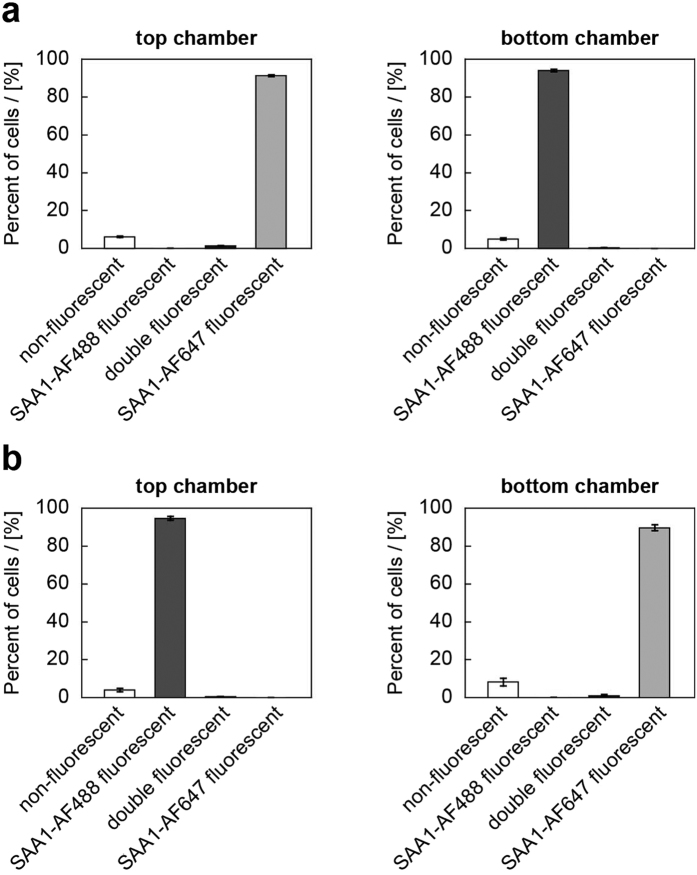
Preventing direct cell-to-cell contacts decreases the transfer efficiency of SAA1 fibrils. Flow cytometric analysis of J774A.1 cells that were preincubated for *t*_*1*_ = 5 h with 0.3 mg/ml non-fibrillar SAA1 and 9 μg/ml fluorescently labelled SAA1 fibrils (either SAA1-AF488 or SAA1-AF647) and then further incubated for *t*_*2*_ = 24 h in one culture plate, but separated by a 0.4 μm membrane. The percentage of double positive cells was quantified based on the flow cytometry scatter plot SAA1-AF488 vs. SAA1-AF647 (upper right area, see Fig. 4a,b, n = 3). (**a**) The cells in the top chamber were preincubated with SAA1-AF647 fibrils, while cells in the bottom chamber were preincubated with SAA1-AF488 fibrils. (**b**) The top chamber contained cells that were preincubated with SAA1-AF488 fibrils while the bottom chamber contained cells that were preincubated with SAA1-AF647 fibrils.

Repeating the flow cytometry experiments with cells loaded with fibrillar SAA1 protein (Fig. 5) also revealed almost no exchange of SAA1 fibrils by the end of the co-incubation period (Fig. 5). Hence, the exchange of SAA1 fibrils and of non-fibrillar SAA1 protein depends strongly on the presence of direct physical contacts between the cells, while an exchange via the medium seems less relevant.

### Increasing the medium volume only moderately affects cell-to-cell transfer

**Figure 6:**
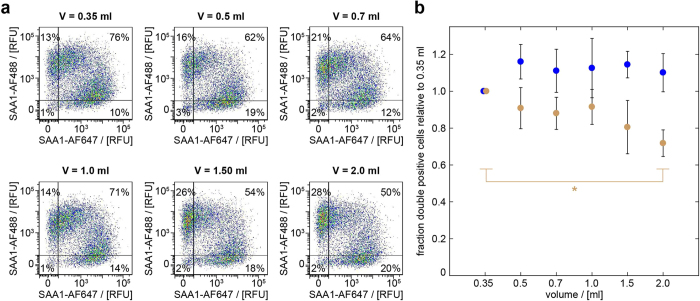
Increasing the volume of the culture medium modestly affects SAA1 transfer. (**a**) Flow cytometric analysis of J774A.1 cells that were preincubated for *t*_*1*_ = 24 h with 1 mg/ml non-labelled SAA1 and 0.02 mg/ml fluorescently labelled SAA1 (either SAA1-AF488 or SAA1-AF647) and co-cultured for *t*_*2*_ = 24 h in wells containing different volumes (V) of cell culture medium as indicated. The vertical and horizontal lines represent the thresholds above cells that were considered as SAA1-AF488 and SAA1-AF647 positive. (**b**) Quantification of the transfer efficiency based on percent of double positive cells in the upper right area of the SAA1-AF488 and SAA1-AF647 scatter plot. Ochre: cells incubated with non-fibrillar SAA1, blue: cells incubated with fibrillar SAA1. The data points at 0.35 ml cell culture volume were set to 1 in each sample series (n = 3, *p < 0.05). RFU: relative fluorescence unit.

This conclusion was further supported by experiments in which cells that were loaded with 1 mg/ml non-labelled SAA1 and either 0.02 mg/ml SAA1-AF488 or SAA1-AF647 (all non-fibrillar), were combined within the same well (in the absence of any membrane) and incubated in the presence of different culture volumes. We hypothesized that transfer through the medium should strongly depend on the medium volume, as was previously suggested for the exchange of mutant superoxide dismutase-1 (SOD1) aggregates between neuronal cells^32^. In the cases of the exchange of non-fibrillar SAA1 protein and of SAA1 fibrils, however, we found only modest, if any, effects of the medium volume. In the case of cells loaded with non-fibrillar SAA1 protein, we found the percentage of double positive cells to decrease by only about 28% as we increased the medium volume from 0.35 to 2 ml (Fig. 6). In the case of cells loaded with SAA1 fibrils there was no discernible decrease of the transfer efficiency within the analysed volume range whatsoever (Fig. 6), providing strong and orthogonal support to our above conclusion that the encountered cell-to-cell transfer depends on physical contacts between the cells. Our observations drastically differ from the ones seen in the aforementioned SOD1 study^32^ which reports a roughly 70% decrease of the double positive cells for a corresponding increase of the medium volume.

### SEM reveals the presence of direct cell-to-cell contacts

**Figure 7:**
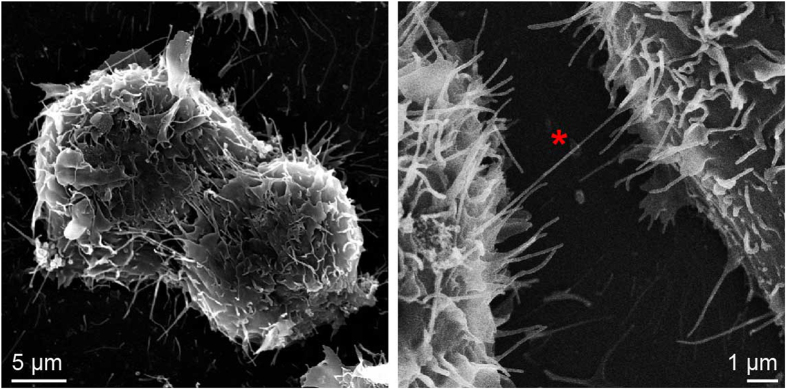
SEM shows direct cell-to-cell contacts. Scanning electron micrograph of cells that were incubated with 1 mg/ml SAA1 protein for *t*_*1*_ = 24 h, and incubated within a new plate for *t*_*2*_ = 24 h to correspond to the incubation conditions used in Fig. 2a. The left image shows extensive direct cell-to-cell contacts. The right image highlights a nanotube (red asterisk).

Finally, we wondered as to whether or not there was any morphological evidence for direct cell-to-cell interactions among the cultured cells and we used scanning electron microscopy (SEM) to analyse cells that were incubated with 1 mg/ml SAA1 protein for an initial period of time *t*_*1*_ of 24 h. The cells were collected by scraping and transferred into a new plate for a further incubation period *t*_*2*_ of 24 h to correspond the experimental conditions in Figs 2 and 3. Analysis of these cells by SEM revealed significant cell-to-cell contacts (Fig. 7) that were defined by substantial lateral interactions of neighbouring cells (Fig. 7, left) as well as by tunneling nanotubes (Fig. 7, right). The observed tunneling nanotubes possessed a width of approximately 50 nm and a length of several micrometres, consistent with reported dimensions of such structures^33^. We conclude that all data imply the transfer of SAA1 protein from cell to cell to strongly depend on direct physical interactions between the cultured cells and that these interactions involve either lateral direct contacts between the cells or tunneling nanotubes.

## Discussion

In this study we show that SAA1 protein can be efficiently transferred between cultured J774A.1 cells that were loaded with different fluorescent variants of SAA1 protein. We further demonstrate that this transfer occurs both with non-fibrillar SAA1 protein as well as with SAA1 fibrils, although the *y*_*max*_ value measured with non-fibrillar SAA1 protein was significantly higher (93%) than that obtained with SAA1 fibrils (46%). Furthermore, the rate constant *k* of exchange was 0.68 h^−1^ with non-fibrillar SAA1 and only 0.13 h^−1^ with SAA1 fibrils. We conclude non-fibrillar SAA1 exchanges faster than SAA1 fibrils and gives rise to a higher percentage of double positive cells within the analysed period of time.

While most previous research on the cell-to-cell transfer of amyloidogenic proteins has focused on brain amyloid diseases, such as Alzheimer’s, Parkinson’s and Huntington’s^24^, much less is known about these processes in systemic amyloidosis. Nevertheless, there has been considerable evidence for the propagation and spreading of these diseases as well. For example, whole body amyloid imaging revealed a successive organ involvement in humans affected by systemic AA, AL and ATTR amyloidosis^30^,^31^,^34^, and there is long-standing evidence that murine AA amyloidosis, the model system underlying the current study, initially involves amyloid deposits within the spleen and only later within kidneys and liver^3^. Murine AA amyloidosis is also the classical example and experimental model system to study prion-like transmission phenomena in systemic amyloidosis. The disease can be propagated between animals by the transfer of AEF-laden macrophages^17^,^35^ or by the oral uptake of AEF-contaminated food products or faeces^36^,^37^.

Our current study now demonstrates SAA1 fibrils and non-fibrillar SAA1 protein to be efficiently transferred between cultured cells. The transfer efficiency appears to be higher, when measured by using flow cytometry compared to confocal microscopy. However, the two methods although recording the same parameter exhibit significant differences in terms of signal sensitivity^38^. Actually, flow cytometry records the fluorescence signal of the whole cell and hence exhibits higher fluorescence sensitivity than confocal microscopy, which eliminates by technical design out-of-focus signals and which is built up serially by individual measurement at every cell location (thereby loosing out-of-focus signals at every single spot). An advantage of confocal microscopy is, of course, the information on the subcellular localization. Anyhow, for the given reasons, flow cytometry as a summary signal will record more emitted fluorescence.

We demonstrate this exchange to largely depend on direct cell-to-cell contacts. Preventing the formation of physical interactions between the cells, for example, by separating the cells with a permeable membrane, thus drastically reduces the exchange of SAA1 protein between separated cell populations (Fig. 4). This conclusion holds true for both non-fibrillar SAA1 protein (Fig. 4) as well as for SAA1 fibrils (Fig. 5). Except from a small drop of exchange efficiency seen with non-fibrillar SAA1 protein (Fig. 6b), there was also no discernible dependence of the transfer efficiency on the medium volume in the range from 0.35 to 2.0 ml. This observation indicates that mechanisms that depend on SAA proteins present in the medium do not strongly contribute to the observed exchange reaction, resembling data obtained for several neurodegenerative amyloid diseases, where there was also evidence for direct physical interactions or tunneling nanotubes between donor and recipient cell^39^,^40^,^41^.

The presently observed exchange scenario hence differs from previously described cell-to-cell transfer reactions which mainly depended on the release of the prion-like agent into the extracellular medium. Examples hereof are fluorescently labelled SOD1 aggregates and their subsequent exchange between neuronal cells^32^, α-synuclein released from cultured SH-SY5Y donor cells^42^, or the transfer of aggregated intracellular Tau protein between C17.2 cells^43^. The observed independence of the exchange on the medium also argues against a strong involvement of exosome-bound SAA1, although there has also been evidence for an association of AEF with exosomes^44^. A passage via exosomes has been suggested, for example, to mediate a cell-to-cell transfer of β-amyloid in Alzheimer’s disease^45^, prion protein^46^, α-synuclein in Parkinson’s disease^47^, and TDP-43 protein from amyotrophic lateral sclerosis^41^.

However, our present study was conducted under conditions where the cultured J774A.1 cells had not formed substantial amounts of extracellular amyloid deposits that would have complicated our present exchange analysis. Such amyloid deposits are only seen in our cultures if the incubation period is prolonged to two or more days in the presence of acute-phase levels of SAA1 protein^22^. It is thus possible that additional exchange mechanisms may participate in the exchange reaction as amyloid deposits emerge in culture.

## Material and Methods

### Recombinant expression and purification of murine SAA1 protein

Murine full-length SAA1.1 protein was recombinantly expressed in *Escherichia coli RV308* cells and purified as described elsewhere^22^. The purified protein was lyophilized and stored at −80 °C until further use.

### *In vitro* fibril formation

SAA1 fibrils were obtained by incubating recombinant SAA1 at 1 mg/ml in 10 mM Tris buffer (pH 8), for 6 days at 37 °C, with continuous shaking (300 rpm). Fibril formation was confirmed by TEM. At the end of the incubation time, sample was concentrated by centrifuging at 16,000 *g* for 30 min at 4 °C and resuspending the pellet in water. Protein concentration was measured by absorbance measurements at 280 nm^48^.

### Unspecific N-fluorescent labelling of murine SAA1 protein

2 mg lyophilized SAA1 protein was dissolved in 500 μl 100 mM sodium carbonate buffer (pH 8). 50 μl of a 4 mg/ml solution of AF488 or AF647 succinimidyl ester (Thermo Fisher Scientific) in dimethyl sulfoxide (Sigma-Aldrich) were added and the solution was incubated at room temperature under continuous shaking (300 rpm) for 1 h in a thermostated mixer (Eppendorf Thermomixer Compact). The labelling reaction was stopped by addition of 100 μl 1.5 M hydroxyl amine (pH 8.5). The solution was centrifuged (16,900 *g*, 30 min) and the pellet was dissolved in 500 μl of a 7.5 M guanidine hydrochloride, 25 mM sodium phosphate buffer (pH 7.4). The supernatant and the pellet-derived solution were separately loaded onto a 3 ml Resource reversed phase chromatography column (GE Healthcare) and eluted using a linear gradient from 0 to 86% (v/v) acetonitrile in 0.1% (v/v) trifluoracetate. The concentration of fluorescently labelled SAA1 protein in the eluted fraction was determined by absorbance at 500 nm (SAA1-AF488, ε = 71,000 M^−1^ cm^−1^) or 653 nm (SAA1-AF647, ε = 239,000 M^−1^ cm^−1^) assuming an average labelling efficiency of one dye group per SAA1 molecule. The proteins purified from supernatant and pellet were lyophilized and stored separately at −80 °C.

### Unspecific N-fluorescent labelling of *in vitro* fibrils

For the unspecific N-fluorescent labelling of the obtained fibrils, an aliquot of fibril sample containing 1 mg of SAA1 protein was diluted to a final volume of 250 μl in 100 mM sodium carbonate buffer (pH 8) and a final concentration of 4 mg/ml SAA1 protein. To this sample, 25 μl of a 4 mg/ml solution of either AF488 or AF647 succinimidyl ester (Thermo Fisher Scientific) in dimethyl sulfoxide were added and incubated under continuous shaking (300 rpm, Eppendorf Thermomixer Compact) at room temperature for 1 h. The coupling reaction was stopped by addition of 100 μl 1.5 M hydroxyl amine solution (pH 8.5) before the sample was dialysed three times against 1 l water using a 3.5 kDa Spectra/Por^®^ 6 Dialysis Membrane (Spectrum Laboratories). The dialysed solution was centrifuged (16,000 *g*, 4 °C, 30 min), and the pellet was resuspended in 100 μl water to estimate protein concentration. The sample was stored at 4 °C until use.

### Transmission electron microscopy (TEM)

A 5 μl aliquot of the sample to be analysed was placed on a carbon-coated copper grid (Electron Microscopy Sciences), incubated for 2 min and washed thrice with 7 μl H_2_O. The grid was stained thrice with 5 μl 2% (w/v) uranyl acetate solution, dried and analysed using a JEM-1400 transmission electron microscope (Jeol), equipped with a 2 k × 2 k TVIPS TemCam-F216 camera.

### ThT binding assay

ThT fluorescence was measured using a LS 55 fluorescence spectrometer (Perkin Elmer). An excitation wavelength of 450 nm was used and the emission spectrum was recorded from 460 to 700 nm. Samples with a volume of 160 μl contained 20 μM ThT, 10 mM Tris buffer (pH 8) and 20 μM freshly dissolved SAA1 protein or SAA1 fibrils as indicated in Fig. 1a. A SUPRASIL^®^ Micro quartz fluorescence cuvette (Type No.: 105.253-QS, Hellma) was used in the measurements. We uniformly accumulated 3 scans per sample, using a scan speed of 100 nm/min and excitation and emission slit settings of 7 nm each. Measurements were performed at room temperature.

### CR binding assay

CR absorbance was measured using a Lambda 35 UV/VIS spectrometer (Perkin Elmer). A sample of 160 μl final volume contained 10 μM CR, 10 mM Tris buffer (pH 8) and 20 μM freshly dissolved SAA1 protein or SAA1 fibrils as indicated in Fig. 1b. All measurements were carried out in a SUPRASIL^®^ Ultra-Micro quartz UV/VIS cuvette (Type No.: 105.201-QS, Hellma) at room temperature. The absorbance spectra were recorded from 200 to 700 nm, using a slit width of 1 nm and a scan speed of 480 nm/min with 1 nm intervals.

### Cell culture

J774A.1 cells (Sigma-Aldrich) were cultivated in Dulbecco’s Modified Eagle Medium (Thermo Fisher Scientific) supplemented with 10% (v/v) heat-inactivated fetal bovine serum (Thermo Fisher Scientific) and 1% (v/v) Antibiotic-Antimycotic solution (Thermo Fisher Scientific) at 37 °C in a humidified atmosphere containing 5% CO_2_. The cells for flow cytometry experiments were usually plated out at a density of 400,000 cells/ml in 24-well plates (Greiner Bio-One) but for LSM in 8-well Nunc^TM^ Lab-Tek^TM^ II slides (Thermo Fisher Scientific). After 24 h, the medium was replaced by fresh medium containing a mixture of 1.0 mg/ml non-labelled SAA1 and 0.02 mg/ml fluorescently labelled SAA1 (either SAA1-AF488 or SAA1-AF647) for an initial time period *t*_*1*_ of 24 h. When cell-to-cell transfer of fibrillar SAA1 was studied, cells were preincubated for an initial time period *t*_*1*_ of 5 h, with 0.3 mg/ml non-fibrillar SAA1 and 9 μg/ml fluorescently labelled SAA1 fibrils (either SAA1-AF488 or SAA1-AF647). Afterwards the medium was removed and the cells were trypsinized with 100 μl Trypsin-EDTA (Merck Millipore) at 37 °C for 5 min. The trypsination reaction was stopped by addition of 500 μl cell culture medium and the cells were scraped off from the plate using a cell scraper (Techno Plastic Products). The cells were collected by centrifugation for 5 min at 200 *g* and room temperature. The cell pellet was resuspended in 500 μl fresh medium. If relevant, cells that had been pretreated with different SAA1 variants in the *t*_*1*_ incubation period, were mixed 1:1, plated out in a new well and either further co-incubated for a time period *t*_*2*_, or further cultured separated by a 0.4 μm membrane (Greiner Bio-One, ThinCerts^TM^, 24 well), as indicated in the experiment.

SAA1 protein was added to the medium of the cells from a stock solution of 10 mg/ml SAA1 dissolved in pure water. Residual trifluoracetate remaining from the purification procedure was removed by filtering this solution twice through a 3 kDa membrane filter (Amicon Ultra-0.5 ml 3 K, Merck Millipore) and centrifugation at 14,000 *g* for 10 min at 4 °C. After each centrifugation step the retentate was filled up to the original volume with pure water and the protein was eluted from the membrane by inversion of the filter and a final centrifugation step at 1,000 *g* for 1 min at 4 °C. Aliquots from the eluted solution were added to the cell medium to reach a final SAA1 concentration of 1 mg/ml. SAA1-AF488 and SAA1-AF647 were dissolved in dimethyl sulfoxide at a concentration of 4 mg/ml and were added to the medium without any filtration at a final concentration of 0.02 mg/ml. SAA1-AF488 and SAA1-AF647 fibrils were added to the medium from a water stock solution of 1 mg/ml.

### Laser scanning microscopy (LSM)

The medium was taken off from the cells to be analysed and the extracellular SAA1 protein remaining in the well was removed by trypsination. This time 50 μl Trypsin-EDTA (Merck Millipore) solution were added and incubated at 37 °C for 3 min. The trypsination reaction was stopped by addition of 350 μl medium, which was immediately replaced with 200 μl phosphate-buffered saline (PBS). Cells were imaged at 37 °C using a LSM710 confocal microscope (Carl Zeiss) and the following excitation/emission settings: 405/410–460 nm (autofluorescence), 488/494–553 nm (SAA1-AF488 fluorescence) and 633/643–695 nm (SAA1-AF647 fluorescence).

### Flow cytometry

For flow cytometric analysis, cells were trypsinized and additionally scraped off from the plate as specified in the general cell culture methods, and then transferred into a fresh 2 ml tube. The cell suspension was centrifuged at 200 *g* for 5 min at 4 °C, after which the supernatant was discarded and the cell pellet was resuspended in 1 ml flow cytometry buffer, consisting of 2 mM EDTA, 0.5% (w/v) bovine serum albumin, 0.1% (w/v) sodium azide in PBS (pH 7.4). This suspension was centrifuged once more and the pellet was resuspended in 200 μl flow cytometry buffer that additionally contained 2% (w/v) paraformaldehyde (Carl Roth) to fix the cells. The cells were incubated in this solution at room temperature for 15 min and centrifuged again at 200 *g*, 4 °C for 5 min. The supernatant was discarded and the pellet was resuspended in 400 μl flow cytometry buffer, transferred into a flow cytometry tube (Sarstedt) and analysed in a BD FACSVerse^TM^ flow cytometer (BD Biosciences) using the following excitation/emission settings: 488/511–543 nm (SAA1-AF488) and 633/655–665 (SAA1-AF647). For each sample 14,000–20,000 events were measured. Data analysis was performed using FlowJo (FlowJo, LLC) software.

### Scanning electron microscopy (SEM)

Cells were grown on sapphire discs (diameter: 3 mm; Engineering Office Wohlwend) and incubated with 1 mg/ml SAA1 protein for an initial time period *t*_*1*_ of 24 h, scraped off the plate and transferred into a new plate where they were incubated for a further time period *t*_*2*_ of 24 h. Afterwards the medium was removed and the samples were incubated at room temperature for 3 h with 100 μl 2.5% (w/v) of glutaraldehyde and 1% (w/v) sucrose in 0.1 M sodium phosphate buffer (pH 7.3). Samples were washed for 2 min with 100 μl PBS and dehydrated in a series of 30% (v/v), 50% (v/v), 70% (v/v) propanol for 5 min each and then for 2 min in 90% (v/v) and for 10 min in 100% (v/v) propanol. The dehydrated samples were critical point dried using a CPD BalTec 030 Critical Point Dryer (Leica) and coated using a Balzers BAF 300 (Bal-Tec) apparatus with 3 nm of platinum. Samples were then analysed using a Hitachi S-5200 scanning electron microscope (Hitachi).

### Protein gel electrophoresis

Proteins were separated on NuPAGE^®^ 4–12% Bis-Tris gradient gels (Thermo Fisher Scientific) using NuPAGE^®^ MES LDS running buffer (Thermo Fisher Scientific). Samples were mixed with 4 x NuPAGE^®^ LDS sample buffer (Thermo Fisher Scientific) and denatured by heating for 10 min at 95 °C. The gels were stained with 2.5 g/l Coomassie brilliant blue R250, 20% (v/v) ethanol and 10% (v/v) acetic acid for 1 h, before they were destained in 30% (v/v) ethanol and 10% (v/v) acetic acid.

### Statistical analysis

Error bars represent the standard deviation and results were analysed by the Student’s t-test (unpaired, unequal variances).

## Additional Information

**How to cite this article**: Claus, S. *et al*. Cell-to-cell transfer of SAA1 protein in a cell culture model of systemic AA amyloidosis. *Sci. Rep.* **7**, 45683; doi: 10.1038/srep45683 (2017).

**Publisher's note:** Springer Nature remains neutral with regard to jurisdictional claims in published maps and institutional affiliations.

## Supplementary Material

Supplementary Information
