# Supplementary material for: Cell-to-cell transfer of SAA1 protein in a cell culture model of systemic AA amyloidosis
Source: Sci Rep. 2017 Mar 31;7:45683. doi: 10.1038/srep45683 (PMC5374501; doi:10.1038/srep45683)
Supplement: Supplementary Information [file srep45683-s1.pdf]

SUPPLEMENTAL INFORMATION

Cell-to-cell transfer of SAA1 protein in a cell culture model of systemic AA amyloidosis

Stephanie Claus[a], Ioana Puscalau-Girtu[a], Paul Walther[b], Tatiana Syrovets[c], Thomas Simmet[c], Christian Haupt[a] and Marcus Fändrich[a*]

[a] Institute of Protein Biochemistry, Ulm University, Helmholtzstr. 8/1, 89081 Ulm, Germany

[b] Central Facility for Electron Microscopy, Ulm University, Albert-Einstein-Allee 11, 89069 Ulm, Germany

[c] Institute of Pharmacology of Natural Products and Clinical Pharmacology, Ulm University, Helmholtzstr. 20, 89081 Ulm, Germany

[*] Correspondence to: Institute of Protein Biochemistry, Ulm University, Helmholtzstr. 8/1, 89081 Ulm, Germany. Tel: +49 731 50-32750, Fax: +49 731 50-32759, E-mail: marcus.faendrich@uni-ulm.de

Supplementary Figure

Figure S1

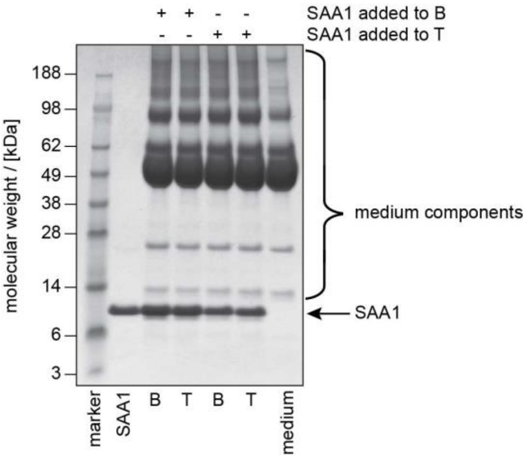

Figure S1

Non-fibrillar SAA1 protein diffuses through the membrane.

Coomassie stained LDS-PAGE gel of cell culture medium that was incubated for 1 day in the bottom (B) or the top chamber (T) as indicated and separated by a 0.4 µm membrane. At time point 0 h 1 mg/ml SAA1 was added to the medium of bottom or top chamber as indicated. The lane labelled SAA1 shows 0.25 mg/ml SAA1 without medium. The lane labelled medium represents cell culture medium without addition of SAA1.
